# Incorporating microbiomes into the One Health Joint Plan of Action

**DOI:** 10.1128/mbio.01456-25

**Published:** 2025-09-08

**Authors:** Estelle Couradeau, Jennifer B. H. Martiny, Fanette Fontaine, Christian Brechot, Marc Bonneville, Karel Callens, the Microbiomes for One Health Initiative, Francesco Asnicar

**Affiliations:** 1 Department CIBIO, University of Trento, Trento, Italy; 2 Department of Chemistry, University of South Florida, Tampa, Florida, USA; 3 Graz University of Technology, Environmental Biotechnology, Graz, Austria; 4 Institut Mérieux, Lyon, France; 5 Institut de Biologie de l'Ecole Normale Supérieure (IBENS), Ecole Normale Supérieure, CNRS, INSERM, Université PSL, Paris, France; 6 Global Virus Network, University of South Florida, Tampa, Florida, USA; 7 Food and Agriculture Organization of the UN, Rome, Italy; 8 The Pennsylvania State University, University Park, Pennsylvania, USA; 9 SRQ Whisky Institute, Sarasota, Florida, USA; 10 Mérieux NutriSciences, Treviso, Italy; 11 NutriOmics, Paris, France; 12 Nutrition Department, Sorbonne Université, Inserm, Pitié-Salpêtrière Hospital, Paris, France; 13 AdventHealth Translational Research Institute, Orlando, Florida, USA; 14 Food and Agriculture Organization of the United Nations, Rome, Italy; 15 School of Hospitality and Tourism Management, University of South Florida, Tampa, Florida, USA; 16 Center for the Advancement of Food Security & Healthy Communities, University of South Florida, Tampa, Florida, USA; 17 King Abdullah University of Science and Technology, Thuwal, Saudi Arabia; 18 College of Marine Science, University of South Florida, St. Petersburg, Florida, USA; 19 Department of Ecology and Evolutionary Biology, University of California, Irvine, Irvine, California, USA; 20 Department of social and environmental medicine, Faculty of Tropical Medicine, IRL HealthDEEP CNRS-Kasetsart University-Mahidol University, Mahidol University, Bangkok, Thailand; 21 Department of Pediatrics, Morsani College of Medicine, University of South Florida, Tampa, Florida, USA; 22 Patel College of Global Sustainability and Department of Integrative Biology, University of South Florida, Tampa, Florida, USA; 23 Agrauxine by Lesaffre, Beaucouzé, France; 24 College of Marine Science, University of South Florida, Tampa, Florida, USA; 25 Lesaffre Institute of Science and Technology, Lesaffre International, Marcq-en-Barœul, France; 26 Sequentia Biotech SL, Barcelona, Spain; 27 Crop Services International, Inc, Marcellus, Michigan, USA; 28 University of South Florida, Tampa, Florida, USA; 29 University of South Florida College of Public Health, Tampa, Florida, USA; 30 The Global Virus Network, Tampa, Florida, USA; 31 USF Center for Microbiome Research, Microbiomes Institute, University of South Florida Morsani College of Medicine, Tampa, Florida, USA; 32 Department of Biochemistry and Microbiology, School of Environmental and Biological Sciences, Rutgers University, New Brunswick, New Jersey, USA; 1 Department of Ecosystem Science and Management, The Pennsylvania State University 171649, University Park, Pennsylvania, USA; 2 Department of Ecology and Evolutionary Biology, University of California 189205, Irvine, California, USA; 3 Food and Agriculture Organization of the United Nations 17107, Rome, Italy; 4 Department of Internal Medicine, University of South Florida 824897, Tampa, Florida, USA; 5 Institut Mérieux 201655, Lyon, France; Georgia Institute of Technology, Atlanta, Georgia, USA

**Keywords:** One Health, microbiome, United Nations

## Abstract

The One Health Joint Plan of Action (2022–2026), developed by the United Nations Quadripartite (FAO, UNEP, WHO, and WOAH), provides a comprehensive framework to address global health risks at the human-animal-plant-environment interface. However, it overlooks the critical role of microbiomes—complex microbial communities that underpin the health of all ecosystems and are central to the One Health paradigm. Microbiomes regulate key processes, such as nutrient cycling, pathogen suppression, antimicrobial resistance (AMR) dynamics, and environmental resilience, making their inclusion essential for achieving One Health goals. We argue that incorporating the central role of microbiomes will help us move from managing the symptoms of these challenges toward addressing their root causes and providing sustainable, long-term solutions. This perspective outlines how microbiome science can enhance the core action tracks of the One Health Plan, offering innovative solutions for zoonotic disease prevention, AMR mitigation, food safety, and environmental sustainability. Integrating microbiomes into the One Health agenda is imperative for fostering proactive, cross-sectoral, and sustainable approaches to global health challenges.

## PERSPECTIVE

One Health is a well-established concept that is now more crucial than ever in tackling modern challenges of zoonotic diseases, antimicrobial resistance, and climate- and environment-related health threats (1). One Health has been described by an international group of experts as “an integrated, unifying approach that aims to sustainably balance and optimize the health of humans, animals, plants, and ecosystems. It recognizes the health of humans, domestic and wild animals, plants, and the wider environment (including ecosystems) are closely linked and interdependent” (OHHLEP) (2).

In 2022, four major international organizations—the Food and Agriculture Organization of the United Nations (FAO), the United Nations Environment Programme (UNP), the World Health Organization (WHO), and the World Organisation for Animal Health (WOAH)—published an overarching plan for combating and preventing major health problems wherever humans, animals, plants, and the environment interface: the One Health Joint Plan of Action (2022–2026) (OHJPA) (3). Working together for the health of humans, animals, plants, and the environment is a comprehensive blueprint for how policies, research programs, and targeted investment can better protect the Earth and its living populations. However, this document does not mention “microbiome,” even though microbiomes play a critical role in achieving each of these goals. This omission reflects the relatively recent emergence of microbiome science and its undervaluation in global health strategies, despite its critical importance. Microbiomes regulate essential processes, such as nutrient cycling, pathogen suppression, antimicrobial resistance dynamics, human health, and environmental resilience, making their inclusion indispensable for achieving the One Health goals.

**Fig 1:**
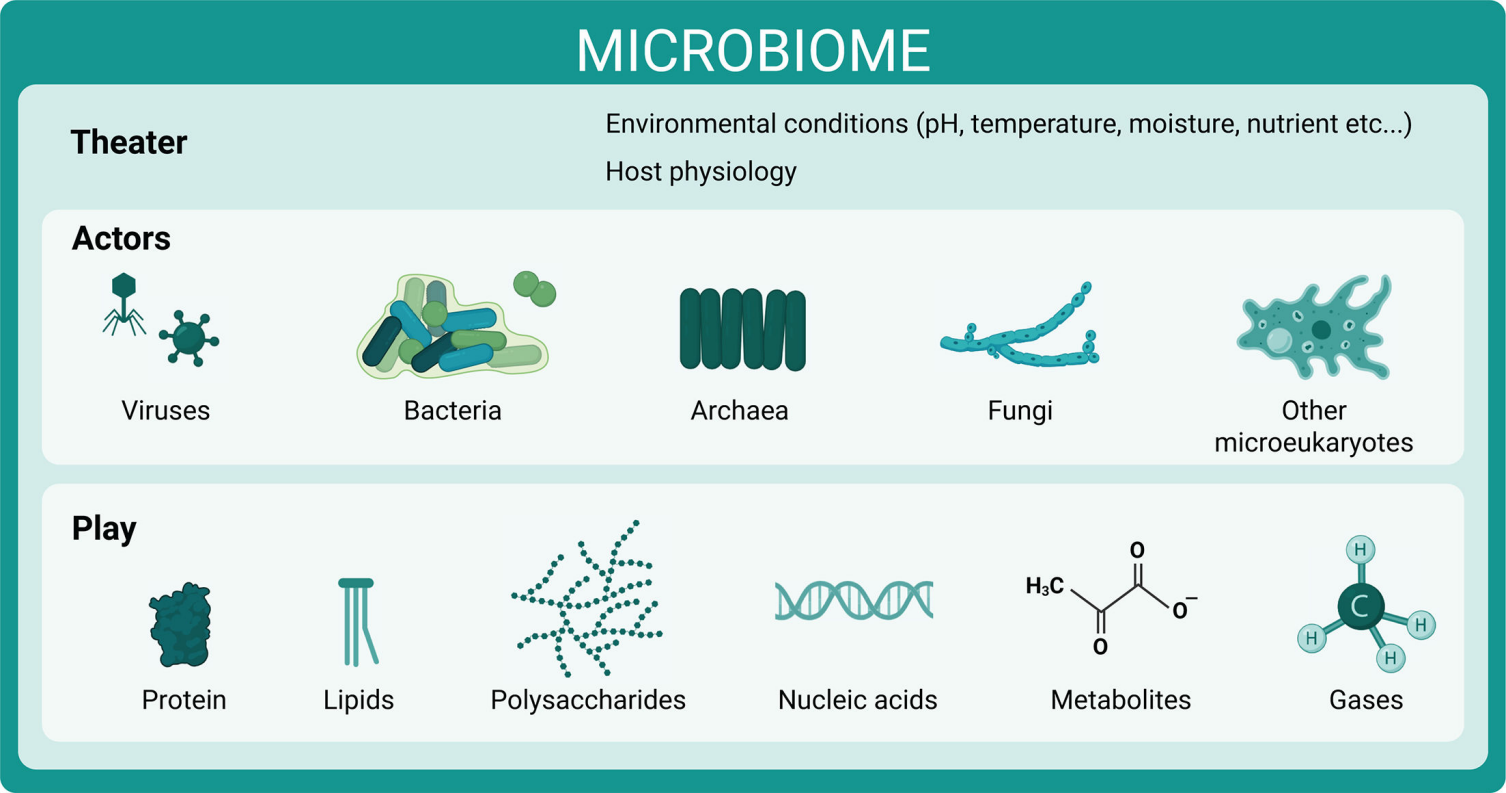
Working definition of a microbiome. Using an extended metaphor, a microbiome can be described as a theater where microorganisms and viruses (actors) perform a play. The environmental conditions and host metabolic status define the theater. The actors are a diverse community composed of viruses, bacteria, archaea, fungi, protists, and algae. The play is the result of their metabolisms and metabolic cross-talks, including synthesis and turnover of biomolecules, toxins and signaling molecules, and processing of wastes, including in the form of potent greenhouse gases (GHG).

Here, we argue that incorporating microbiomes into the six action tracks outlined in the OHJPA will strengthen the framework’s ability to address global health challenges. Microbiomes are communities of microorganisms (bacteria, archaea, fungi, and other microeukaryotes) and viruses that inhabit various hosts and environments and support their functions, thanks to their diversity and metabolic versatility (4, 5) (Fig. 1). This essay outlines how microbiome science can enhance the plan’s objectives and propose a set of actionable recommendations to ensure microbiomes are fully integrated into the One Health agenda.

## MICROBIOMES ARE CRUCIAL INTEGRATORS ACROSS ONE HEALTH SYSTEMS

**Fig 2:**
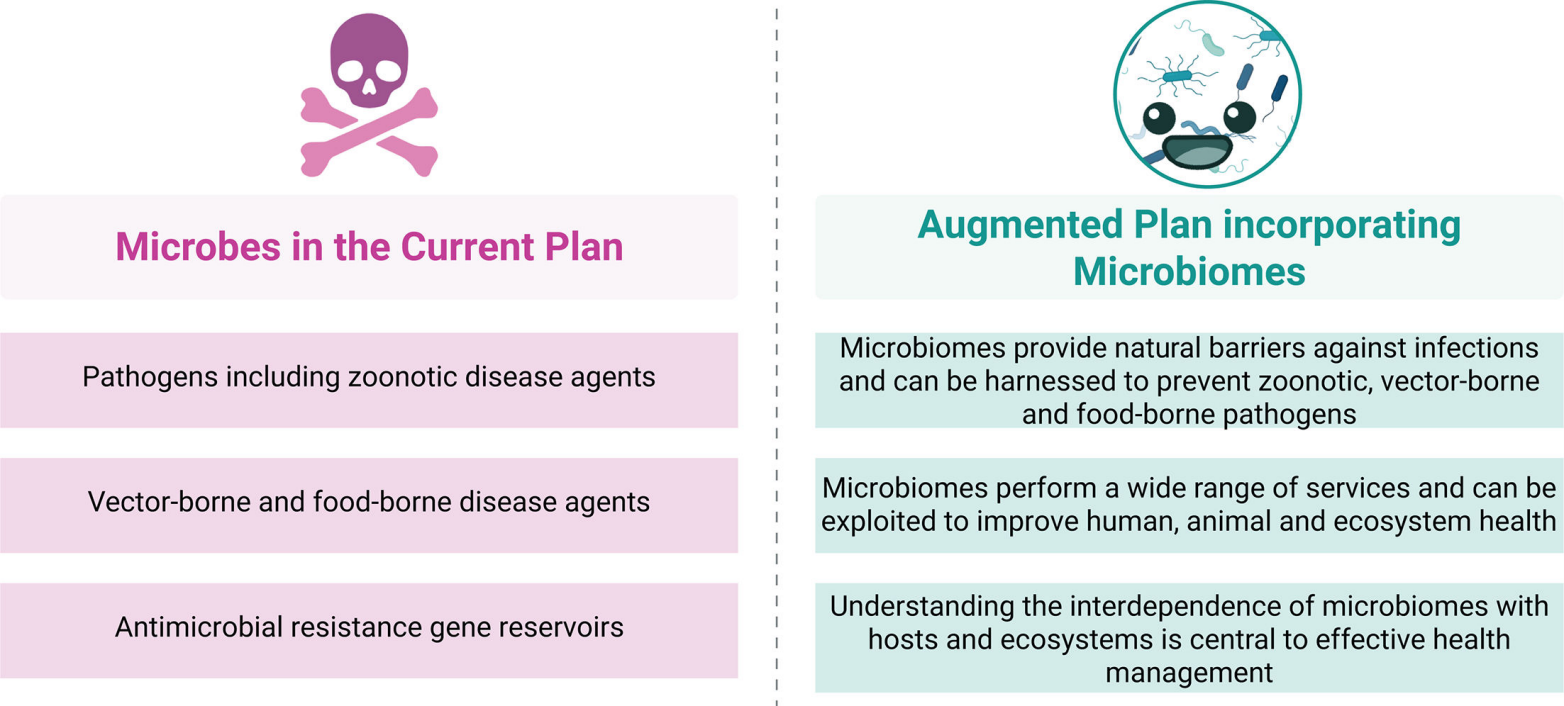
Integration of microbiomes in the One Health Joint Plan of Action. The narrative of the current One Health Plan of Action (left) views microbes through a pathogen lens, whereas broadening this perspective by considering microbiomes (right) is essential for One Health management.

To define a microbiome, the conceptual diagram in Fig. 1 draws a parallel with a theater play (5). Microbiomes are so ubiquitous that their activities penetrate and integrate the subjects of all the primary goals of the OHJPA (3). Figure 2 provides a contrasting comparison showing the narrow focus of microbial integration in the OHJPA through the lens of risk and how augmentation with a microbiome perspective would enhance the current plan. The current plan misses three areas where microbiomes, as communities of cohabiting microorganisms, can play an inherent part of the One Health solutions: (i) microbiomes, which include beneficial and harmless microorganisms, as well as pathogenic ones, can keep pathogens in check when the microbiome is in balance (eubiosis) and can lead to disease when out of balance (dysbiosis); (ii) microbiomes perform essential functions to support human, animal, and plant life and broader ecosystem functioning; and (ii) recognizing the interdependent nature of microbiomes across hosts and ecosystems is central to the management of human and environmental health and resilience.

Microbiomes and microbial products connect host-environment compartments in tangible ways, thanks to their ability to move across reservoirs (6, 7). However, microbiomes are typically studied within isolated reservoirs, the two main classes being host-associated microbiomes and the larger environmental microbiomes in the context of the ecosystems where they live. A host-associated microbiome is a collection of microbes that reside in and on hosts, such as humans, plants, or animals. They typically form symbiotic relationships with the hosts, helping with digestion, immune regulation, and nutrient absorption. They can also play protective roles in the case of infection by pathogens. For example, the effect of *Clostridioides difficile* on human gut health or severity of COVID-19 infection depends on the gut microbiome’s overall health (8, 9). In another example, the effectiveness of vaccines on animals varies with the condition of the host’s immune system and the microbiomes that support it (10, 11). More broadly, a deep understanding of disease transmission, containment, and prevention requires knowledge of how microbiomes travel across species through host-to-host transmission and how the success of this transport depends on the “health” of other host microbiomes.

The second class of microbiomes, environmental microbiomes, inhabit all ecosystems on the planet, including soil, water, and air. Environmental microbiomes play critical roles in regulating ecological processes, such as nutrient cycling, decomposition, carbon sequestration, pollutant degradation, and production and consumption of greenhouse gases. Thus, robust microbiomes maintain the health and balance of ecosystems, supporting biodiversity, soil fertility, water quality, and plant and animal growth.

**Fig 3:**
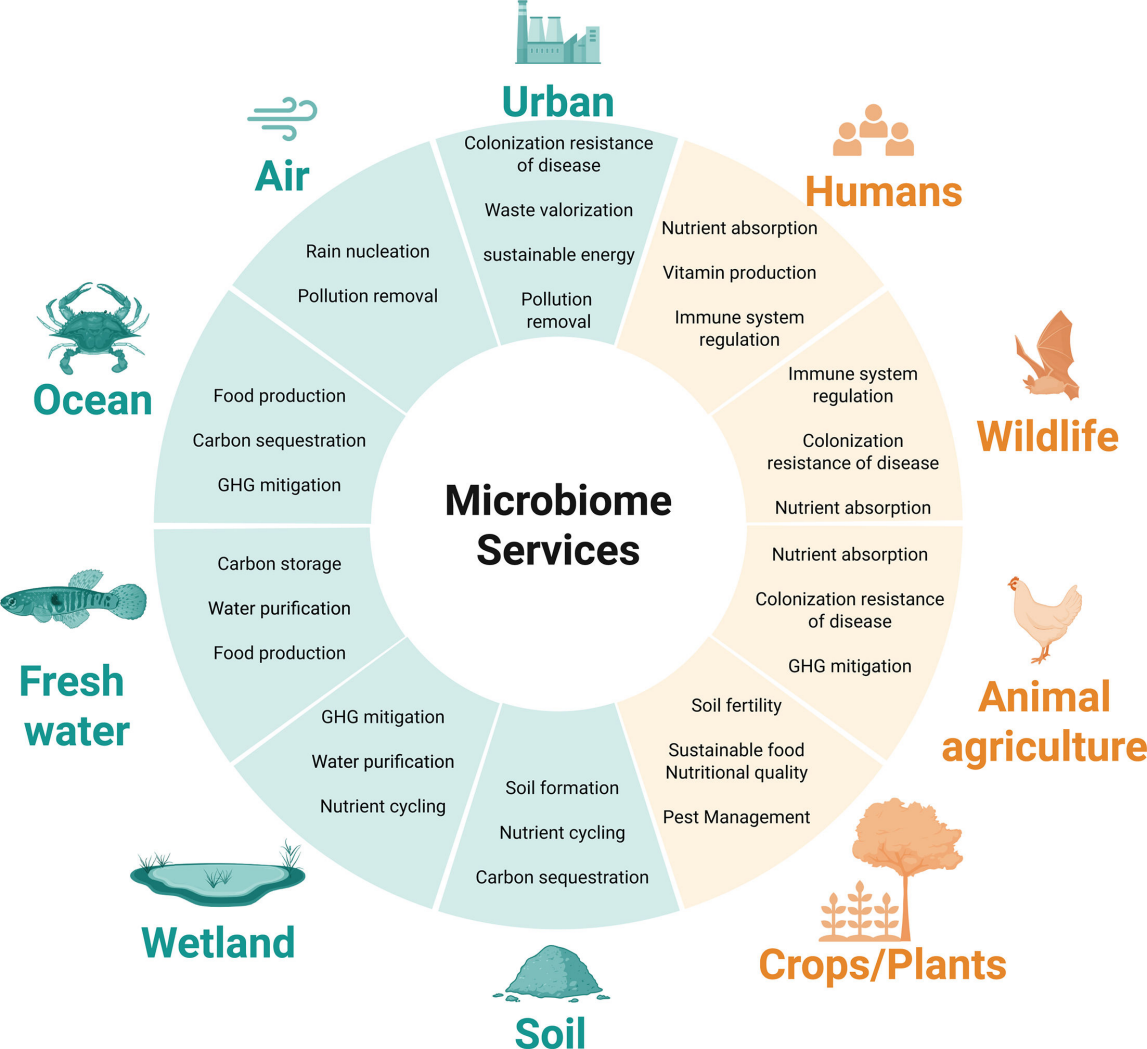
Microbiome services. Microbiomes provide services to their host (orange) and to the environment they inhabit (green). GHG, greenhouse gases. This list is non-exhaustive; see also reference 14.

Microbiomes provide a wide range of services to all managed and natural ecosystems, as summarized in Fig. 3. Multiple microbiome services functionally link many ecosystems, from the human gut to forest soils to river health (6, 7). For example, microbiome nitrogen cycling function (or dysfunction) in soils can affect microbiomes and other biological entities in downstream waterways, affecting the chain of life from river to ocean to human (12, 13).

## USING MICROBIOME KNOWLEDGE TO STRENGTHEN ONE HEALTH PLANS

**Fig 4:**
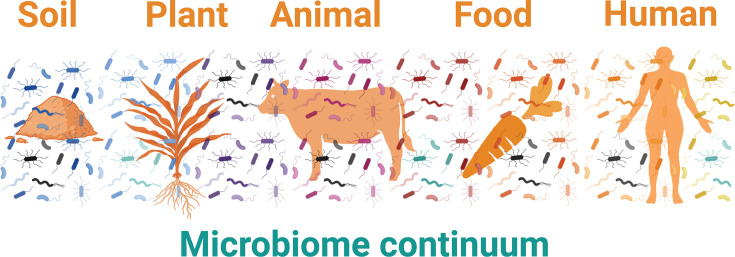
The environment-host microbiome continuum. Illustration of the microbiome continuum in the soil to human One Health thread, mediated by food production and consumption. Microbes experience the world as a continuum; they can move and encounter other microbiomes through natural processes and anthropogenic management.

Incorporating microbiomes in the One Health concept presents numerous opportunities for sharing experiences and creating synergies between stakeholders working in different sectors. This approach is grounded in the idea that similar microbiome-based mechanisms and paradigms may underpin health in different host or environmental settings. In particular, the health of host and environment microbiomes is determined by similar parameters (such as microbiome structural or functional diversity and redundancy, which are associated with increased resilience) that can be monitored using similar molecular tools (15). Microbiomes serve as tangible connectors between environmental, animal, and human systems, forming a microbiome continuum (Fig. 4) that can be strategically leveraged to craft One Health solutions (6). For instance, the soil microbiome plays a pivotal role in nutrient cycling and plant health, directly influencing the nutritional quality of plant-based foods (16). When animals consume plants, some plant-associated microbes (endophytes) interact with their gut microbiomes (17). Similarly, the food humans eat contains microbes that interact with their gut microbiomes (18). Human interventions, such as applying animal manure to soil or tilling fields, further influence soil microbial activity, creating downstream effects throughout the entire food chain (14, 19).

Understanding these interconnected microbiome processes is essential for predicting the dispersion, filtering, and epidemiology of pathogens and other detrimental microbes within these communities. By analyzing how microbiomes move through and connect environmental, animal, and human compartments, we can design more effective One Health strategies that address root causes rather than symptoms.

**Fig 5:**
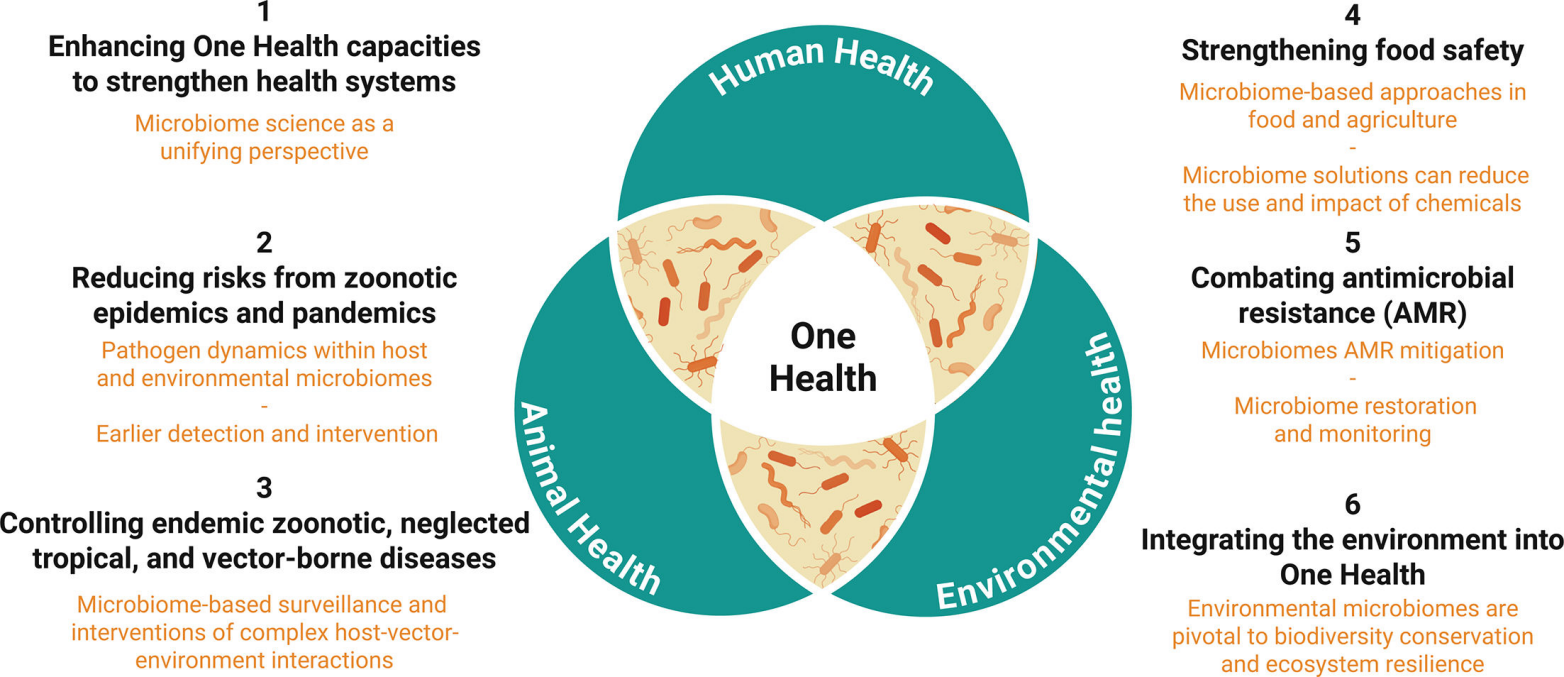
Microbiome contributions to the six tracks of the One Health plans. Microbes tangibly connect (overlapping Venn diagram sections) the three focus areas of One Health (center panel). This figure summarizes the six tracks of the ONJPA and how microbiome science can enhance them.

The OHJPA breaks down its objectives into six action tracks (3). Figure 5 shows how microbiomes can contribute to these objectives.

### Action track 1: enhancing One Health capacities to strengthen health systems

To build effective One Health systems, integrating microbiomes as a core concept is critical. The framework should move from a species-focused perspective to a “biomes” perspective, recognizing the interconnected roles of microbiomes in human, animal, plant, and environmental health. This reframing can inform institutional policies, training programs, and governance structures. For example, curriculum development can include the role of microbiomes in nutrition, disease dynamics, and environmental sustainability. As microbiome science matures into its own field and training programs (20), curricula can be tailored to these relevant fields. For instance, a promising initiative is the inclusion of microbiome science in public health training programs, where professionals learn to monitor environmental microbiomes for early signs of disease outbreaks or ecosystem degradation.

International collaboration is also vital. For instance, efforts to integrate microbiome data into global databases, such as the WHO’s Global Health Observatory or the Global Biodiversity Information Facility (GBIF), could enhance data-sharing and surveillance capacities. Investments in microbiome research, particularly in low- and middle-income countries (LMICs), can strengthen local health systems, allowing them to predict and respond to emerging challenges more effectively. By embedding microbiome knowledge into institutional frameworks, Track 1 can lay the foundation for the remaining action tracks, ensuring health systems are equipped to address the complexities of One Health.

### Action track 2: reducing the risks from emerging and re-emerging zoonotic epidemics and pandemics

Emerging zoonotic diseases often arise at the human-animal-environment interface, where microbiomes serve as critical mediators. Microbiome-based surveillance systems can identify microbial shifts that signal potential spillover events (21). For example, research has shown that environmental stressors such as deforestation can alter soil and water microbiomes, increasing the survival of zoonotic pathogens like *Leptospira* in contaminated water sources (22, 23). Monitoring these shifts can provide early warning signals for public health interventions.

Predictive modeling based on microbiome dynamics can also reveal critical pathways for disease emergence. For instance, during the COVID-19 pandemic, studies suggested that changes in the gut microbiome influenced immune responses to the virus. In-depth studies are still needed to better understand the actual impact of gut dysbiosis vs. eubiosis as well as the mechanisms involved; moreover, most of the available studies on this topic are based on correlation and fail to demonstrate causal association (24–26). However, despite these limitations, system-wide monitoring of microbiomes across hosts and environments could have provided valuable insights into disease progression and transmission risks. By incorporating microbiome science into zoonotic disease prevention strategies, Track 2 can enhance early detection and containment efforts, ultimately reducing the frequency and severity of pandemics.

### Action track 3: controlling and eliminating endemic zoonotic, neglected tropical, and vector-borne diseases

Microbiome science offers innovative solutions for controlling endemic and vector-borne diseases, through inhibition of pathogen development in the vector or blockade of transmission to humans. For instance, the introduction of *Wolbachia* secies bacteria into mosquito populations has proven effective in reducing the transmission of arboviral diseases such as dengue and Zika. *Wolbachia* spp. reduce mosquito lifespans and interfere with virus replication, providing a sustainable, microbiome-based alternative to chemical insecticides. This approach has been successfully deployed in regions such as northern Australia, where dengue cases have dramatically declined (27). Many other microbiome-based strategies have been proposed to tackle vector-borne diseases, such as paratransgenesis, probiotic baiting, or microbiome engineering for transmission blocking (28, 29), whose effectiveness and impact remain to be formally assessed through appropriate entomological, ecological, and epidemiological indicators.

Additionally, microbiome-based surveillance systems can detect early shifts in microbial populations that signal increased disease risks. For example, monitoring livestock microbiomes could help farmers identify dysbiosis linked to diseases, such as brucellosis or avian influenza (30–33). Community-centric education programs can further promote microbiome-friendly practices, such as reducing pesticide use and adopting sustainable farming techniques. By integrating microbiome science into disease control strategies, Track 3 can reduce disease burdens while promoting ecological and public health resilience.

### Action track 4: strengthening the assessment, management, and communication of food safety risks

Microbiomes play a central role in food safety, influencing everything from soil health to contamination risks. For example, bioremediation techniques using beneficial microbes can reduce mycotoxin contamination in crops. *Bacillus subtilis*, a bacterium, has been shown to inhibit the growth of *Aspergillus* secies fungi, which produce aflatoxins harmful to human and animal health. This approach has been successfully tested to inhibit *A. flavus* in soil and peanuts and decrease aflatoxin B1 content in seeds (34). Educational programs targeting farmers and food producers can further promote microbiome-friendly practices, such as reducing the use of chemical fertilizers and pesticides in favor of organic alternatives. For consumers, a “microbiome-friendly” food label—similar to organic certification—could increase awareness and demand for safe, sustainable food products. Governments can also incorporate microbiome health indicators, such as the absence of antimicrobial resistance (AMR) genes of medical concern, into food safety regulations, ensuring a holistic approach to managing contamination risks. By leveraging microbiome science, Track 4 can improve food safety while supporting sustainable agricultural practices.

### Action track 5: curbing the silent pandemic of AMR

AMR is a growing global health threat, and microbiomes are at the heart of its dynamics. For example, excessive antibiotic use in agriculture has led to the proliferation of AMR genes in soil and water microbiomes, which can transfer to human pathogens (35). A promising solution to decrease the use of antibiotics involves using probiotics or beneficial microbes to outcompete harmful bacteria, reducing the need for antibiotics in livestock farming (36). For example, the supplementation of broiler feed with *Saccharomyces cerevisiae* or *Lactobacillus acidophilus* appears as an effective antibiotic-free alternative to the widely used zinc-bacitracin (ZnB) in poultry farming (37).

Microbiome-based monitoring systems can also track AMR markers across clinical, agricultural, and environmental settings, providing critical data for mitigating resistance (38). For instance, metagenomic sequencing can identify hotspots of AMR gene transfer, enabling targeted interventions. Policies that incentivize microbiome-friendly antimicrobial strategies, such as phage therapy or precision antibiotics, are essential for preserving antimicrobial efficacy (39). By integrating microbiome science into AMR strategies, Track 5 can curb silent pandemics and protect global health.

### Action track 6: integrating the environment into One Health

Environmental microbiomes are critical for ecosystem health, biodiversity, and climate resilience. For example, soil microbiomes play a key role in carbon sequestration, with microbes converting organic matter into stable soil carbon (40). Practices like reduced tillage and organic fertilization can enhance soil microbial diversity, increasing carbon capture efficiency (41). A notable example is the use of microbial inoculants in agriculture with the intent to partially replace synthetic fertilizers and pesticides in the next decades (42, 43).

Microbes also have potential in bioremediation, such as breaking down plastic waste in oceans or degrading pesticides in agricultural soils. For instance, the bacterium *Ideonella sakaiensis* can degrade polyethylene terephthalate (PET), offering a microbiome-based solution to plastic pollution (44). Integrating microbiome science into global biodiversity and climate frameworks, such as the Paris Agreement, could amplify these efforts. By leveraging microbiomes, Track 6 can promote sustainable environmental management and mitigate climate change impacts.

## INTEGRATING MICROBIOMES INTO ONE HEALTH: STAKEHOLDER ROLES AND RECOMMENDED ACTIONS

The successful integration of microbiome science and innovation into the One Health Joint Plan of Action (OHJPA) requires clearly defined roles across a diverse range of stakeholders. Policymakers and government agencies play a foundational role by embedding microbiome considerations into legal and regulatory frameworks. This includes streamlining current regulation and working towards a globally harmonized framework for microbiome-based interventions. For instance, the laws applying to the regulation of probiotics and microbiota transplants in humans and the environment vary greatly between countries (45). In the United States, the release of engineered and non-engineered microbial species is evaluated for their impact on human and environmental health (Microbial Pesticides Data Requirements [46]), but there is no consensus on how to quantify these impacts (47). There is also a governmental role in mandating microbiome literacy and training for public sector professionals and ensuring that microbiome science and innovation remain apolitical and grounded in evidence.

International organizations, particularly the Quadripartite (FAO, WHO, UNEP, WOAH), are uniquely positioned to coordinate global action. They are called upon to lead efforts in aligning microbiome-relevant policies, setting international standards, and facilitating capacity-building—especially in low- and middle-income countries. These organizations can also help integrate microbiome indicators into global One Health-related surveillance and monitoring systems, ensuring alignment with existing biodiversity, climate, and other health goals.

Health, agriculture, and environmental professionals are critical on-the-ground actors. Their responsibilities include promoting practices that support beneficial microbiome functions, as well as implementing microbiome-based solutions for disease prevention and dysbiosis management. In parallel, researchers and the scientific community must work to standardize microbiome research methods and propose measurable One Health indicators to monitor efficacy and impact of microbiome-based strategies, incorporate microbiome data into surveillance databases, and develop new tools for early detection and risk assessment across human, animal, and environmental systems.

Education institutions and capacity-building organizations are essential in developing curricula, degrees, and training programs that foster a new generation of interdisciplinary microbiome-literate professionals. Stakeholder uptake of microbiome innovations requires that they are socially acceptable and impactful in diverse local contexts. This implies culturally sensitive community engagement, inclusive co-design processes, citizen science programs, and microbiome-friendly labeling tailored to diverse cultural contexts. Public literacy campaigns can further strengthen this foundation among practitioners and the public. The private sector also plays a vital role in scaling innovation by partnering in research and promoting sustainable products and practices that support microbiome health. The public sector should ensure that socio-cultural and behavioral considerations are embedded in policy design to ensure sustained impact.

Low- and middle-income countries (LMICs) are at the same time highly vulnerable to One Health threats but also rich in microbial diversity. While they should be at the center of One Health strategies, their infrastructure for sequencing, bioinformatics, and technical research capacities remains limited. Beyond strengthening existing local initiatives while integrating these skills and infrastructures, a phased rollout of projects in priority areas, such as soil health or water-borne disease surveillance, could accelerate the implementation of microbiome-based approaches in LMICs (48, 49).

Finally, increased and sustained funding from donors and development partners is crucial to support these efforts. Investment in microbiome science and innovation, particularly in LMICs, will accelerate research, strengthen surveillance, and unlock scalable solutions aligned to facilitate progress towards the One Health goals. Taken together, this coordinated, stakeholder-specific approach ensures that microbiome integration is both actionable and transformative across the One Health agenda.
